# Supplementary material for: Fostering Prevention of Cervical Cancer by a Correct Diagnosis of Precursors: A Structured Case-Based Colposcopy Course in Finland, Norway and UK
Source: Cancers (Basel). 2020 Oct 30;12(11):3201. doi: 10.3390/cancers12113201 (PMC7692698; doi:10.3390/cancers12113201)
Supplement: Supplementary file 1 [file cancers-12-03201-s001.zip › cancers-953396-supplementary file 1.pdf]

Supplementary File 1: EFC Basic course

Course design

Overall goal

The participant knows basic colposcopical methodology and can apply that in a clinical setting

Structure

1-day course: 3x 1.5 – 2.5-hour sessions (total 360min)

As part of the introduction there is an initial session which aims to indicate the learning objectives of the course and also serves as a baseline assessment.

The sessions include lectures on the theoretical background of colposcopy and the principles of colposcopic management. In the end of each lecture there is a problem-solving session focusing on image recognition and case management.

The interactive problem-solving sessions include a number of images that randomly chosen participants are asked to analyse. If the participant has problems solving the case the teacher supports the process by asking questions and giving hints and thus stepwise bringing the structured analysis forward.

Lectures

Introduction

Goal

The participant is familiar with the program and design of the course and gets engaged in the course.

Instructional objectives for the lecturer

Describe and explain the design of the course

Set ground rules for problem-solving sessions

Undertake initial course assessment

Session 1- Colposcopic principles

Goal

The participant is able to describe the cytology and histological morphology of the normal cervix; identify the normal findings and illustrate them in a colposcopic image; know the key issues needed for a successful colposcopic examination and history and can apply the information in a clinical setting

Learning objectives

After the lecture the participant can:

- list the key questions that must be addressed in any colposcopic examination
- choose the important issues and questions needed to take a relevant patient history
- classify the different types of transformation zone (TZ), understands its significance and identify the location and type of the TZ in a colposcopical image
- describe the normal cervical cytological and histological morphology (including metaplasia and TZ)
- recognize normal findings (including effects of age and pregnancy) in a colposcopical image

Session 2 – Colposcopic diagnosis

Goal

The participant can describe the cytology and histological morphology of the abnormal cervix; identify the features of abnormal findings and illustrate them in a colposcopic image; report the basic principles of diagnostic principles of colposcopy and apply them in a clinical setting.

Learning objectives

After the lecture the participant can:

- recognize and describe common non-neoplastic pathology
- recognize and describe the features of CIN, cGIN and VAIN and identify these in a colposcopical image
- recognize and describe the features of cervical cancer and identify these in a colposcopical image
- apply the RCI or Swede index when analysing the findings in a colposcopic image
- demonstrate the key steps in colposcopic decision making
- apply these skills to a clinical setting

Session 3 – Colposcopic management

Goal

The participant can describe the role of colposcopy in the management of CIN; recall the European guidelines of colposcopic management and apply the knowledge in a clinical setting.

Learning objectives

After the lecture the participant can:

- recall European guidelines of colposcopic management and plan patient treatment or follow-up based on these guidelines
- explain the relative value of colposcopy in relation to cytology and clinical setting and apply this when analysing a patient case
- describe the influence of colposcopic findings on management
- describe the different types of biopsy (CDB, simple excisional, extended) and choose the optimal biopsy type for different colposcopical findings

- apply the knowledge to prepare a plan for management of colposcopic abnormality
