# Supplementary material for: Fostering Prevention of Cervical Cancer by a Correct Diagnosis of Precursors: A Structured Case-Based Colposcopy Course in Finland, Norway and UK
Source: Cancers (Basel). 2020 Oct 30;12(11):3201. doi: 10.3390/cancers12113201 (PMC7692698; doi:10.3390/cancers12113201)
Supplement: Supplementary file 1 [file cancers-12-03201-s001.zip › cancers-953396-supplementary file 2.docx]

Supplementary File 2 Questionnaire 2 ID number: ____

**ColpoEdu 2015 picture test**

Each picture will be shown 30 seconds. For each picture choose the appropriate junction type and diagnosis. Also mark on the VAS scale how confident you feel about your answer.

This is pre-test

post-test

**Picture 1.**

SCJ type T1 T2 T3

Diagnosis

a) normal

b) CIN 1

c) CIN 2

d) CIN 3

e) microinvasive squamous cancer

f) SCC (scuamous cell carcinoma)

g) CGIN (glandular atypia)

h) Adeno carcinoma

How confident do you feel about your answer?

I---------------------------------------------------------------------------------I

not at all confident very confident

**Picture 2.**

SCJ type T1 T2 T3

Diagnosis

a) normal

b) CIN 1

c) CIN 2

d) CIN 3

e) microinvasive squamous cancer

f) SCC (scuamous cell carcinoma)

g) CGIN (glandular atypia)

h) Adeno carcinoma

How confident do you feel about your answer?

I---------------------------------------------------------------------------------I

not at all confident very confident

**Picture 3.**

SCJ type T1 T2 T3

Diagnosis

a) normal

b) CIN 1

c) CIN 2

d) CIN 3

e) microinvasive squamous cancer

f) SCC (scuamous cell carcinoma)

g) CGIN (glandular atypia)

h) Adeno carcinoma

How confident do you feel about your answer?

I---------------------------------------------------------------------------------I

not at all confident very confident

**Picture 4.**

SCJ type T1 T2 T3

Diagnosis

a) normal

b) CIN 1

c) CIN 2

d) CIN 3

e) microinvasive squamous cancer

f) SCC (scuamous cell carcinoma)

g) CGIN (glandular atypia)

h) Adeno carcinoma

How confident do you feel about your answer?

I---------------------------------------------------------------------------------I

not at all confident very confident

**Picture 5.**

SCJ type T1 T2 T3

Diagnosis

a) normal

b) CIN 1

c) CIN 2

d) CIN 3

e) microinvasive squamous cancer

f) SCC (scuamous cell carcinoma)

g) CGIN (glandular atypia)

h) Adeno carcinoma

How confident do you feel about your answer?

I---------------------------------------------------------------------------------I

not at all confident very confident

**Picture 6.**

SCJ type T1 T2 T3

Diagnosis

a) normal

b) CIN 1

c) CIN 2

d) CIN 3

e) microinvasive squamous cancer

f) SCC (scuamous cell carcinoma)

g) CGIN (glandular atypia)

h) Adeno carcinoma

How confident do you feel about your answer?

I---------------------------------------------------------------------------------I

not at all confident very confident

**Picture 7.**

SCJ type T1 T2 T3

Diagnosis

a) normal

b) CIN 1

c) CIN 2

d) CIN 3

e) microinvasive squamous cancer

f) SCC (scuamous cell carcinoma)

g) CGIN (glandular atypia)

h) Adeno carcinoma

How confident do you feel about your answer?

I---------------------------------------------------------------------------------I

not at all confident very confident

**Picture 8.**

SCJ type T1 T2 T3

Diagnosis

a) normal

b) CIN 1

c) CIN 2

d) CIN 3

e) microinvasive squamous cancer

f) SCC (scuamous cell carcinoma)

g) CGIN (glandular atypia)

h) Adeno carcinoma

How confident do you feel about your answer?

I---------------------------------------------------------------------------------I

not at all confident very confident

**Picture 9.**

SCJ type T1 T2 T3

Diagnosis

a) normal

b) CIN 1

c) CIN 2

d) CIN 3

e) microinvasive squamous cancer

f) SCC (scuamous cell carcinoma)

g) CGIN (glandular atypia)

h) Adeno carcinoma

How confident do you feel about your answer?

I---------------------------------------------------------------------------------I

not at all confident very confident

**Picture 10.**

SCJ type T1 T2 T3

Diagnosis

a) normal

b) CIN 1

c) CIN 2

d) CIN 3

e) microinvasive squamous cancer

f) SCC (scuamous cell carcinoma)

g) CGIN (glandular atypia)

h) Adeno carcinoma

How confident do you feel about your answer?

I---------------------------------------------------------------------------------I

not at all confident very confident

**Thank you for your participation!**
