# Supplementary material for: Fostering Prevention of Cervical Cancer by a Correct Diagnosis of Precursors: A Structured Case-Based Colposcopy Course in Finland, Norway and UK
Source: Cancers (Basel). 2020 Oct 30;12(11):3201. doi: 10.3390/cancers12113201 (PMC7692698; doi:10.3390/cancers12113201)
Supplement: Supplementary file 1 [file cancers-12-03201-s001.zip › cancers-953396-supplementary file 3.pdf]

Supplementary File 3: Questionnaire

ID number: ______

ColpoEdu 2015 – general information and written pre-test

Fill in this form before the start of the lecture and give it to the staff together with the forms of the picture pre-test. Choose the appropriate answer or mark the answer on the VAS scale with a

General information

1.Gender female male

2. Age ______years

3. How many years of experience in Obstetrics & Gynaecology do You have?

4. Experience of colposcopies (amount done) 0 – 50 50-100 100-200 >200

5. How many years have you done colposcopies? ______ **years**

6. How many colposcopies do you do in a year (amount)? 0 – 50 50-100 100-200 >200

7. Have You attended an earlier course or have You had other colposcopy education?

You can choose more than one alternative

- O Online course, which? ____
- O Book, which? ____
- O Hands-on training, where? ____
- O Lecture / clinical course, which? ____________________________________

Study ID number:_________

Written pre-test

Please describe your colposcopic management in the following cases . Please choose an option for each scenario from options 1 to 10 below. You may choose more than 1 option. Please mark as well on the VAS scale how confident you feel about your answer.

Options

1. no cytology/ biopsy but repeat colposcopy at a later date
2. repeat cytology at a later date
3. repeat cytology and colposcopy at a later date
4. endocervical curettage
5. punch biopsy x1
6. more than 1 punch biopsy
7. excisional biopsy
8. ablative treatment
9. other (please describe)
10. I don't understand

Assume unless otherwise stated that all patients are 25 years old and nulliparous

Case 1. Patient has referral cytology of LSIL, her colposcopy was satisfactory and revealed a low grade acetowhite lesion confined to the ectocervix. 2 colposcopically directed punch biopsies revealed CIN 1 with HPV change. Which option or options would you recommend for further management?

Answer: __________

How confident do you feel about your answer?

I--I

not at all confident   very confident

Case 2. Patient has referral cytology of LSIL, her colposcopy was satisfactory and revealed a low grade acetowhite lesion confined to the ectocervix. 2 colposcopically directed punch biopsies revealed CIN 3. Which option or options would you recommend for further management?

Answer: __________

How confident do you feel about your answer?

I--I

not at all confident   very confident

Case 3. Patient has referral cytology of LSIL, her colposcopy was satisfactory and revealed a high grade acetowhite lesion confined to the ectocervix. Which option or options would you recommend for further management?

Answer: __________

How confident do you feel about your answer?

I--I

not at all confident   very confident

Case 4. Patient has referral cytology of atypical glandular cells (endocervical cells favour neoplasia), her colposcopy was satisfactory and normal. She is 45 years old. Which option or options would you recommend for further management?

Answer: __________

How confident do you feel about your answer?

I--I

not at all confident   very confident

Case 5. Patient has referral cytology of HSIL, her colposcopy was satisfactory and revealed a high grade acetowhite lesion confined to the ectocervix. Which option or options would you recommend for further management?

Answer: __________

How confident do you feel about your answer?

I--I

not at all confident   very confident

Case 6. Patient has referral cytology of HSIL, her colposcopy was unsatisfactory and revealed a high grade acetowhite lesion confined to the cervix. She is 45 years old. Which option or options would you recommend for further management?

Answer: __________

How confident do you feel about your answer?9

I--I

not at all confident   very confident

Case 7. Patient has referral cytology of HSIL, her colposcopy was satisfactory and revealed a low grade acetowhite lesion confined to the ectocervix. Which option or options would you recommend for further management?

Answer: __________

How confident do you feel about your answer?

I---I

not at all confident very confident

Case 8. Patient has referral cytology of HSIL, her colposcopy was satisfactory and revealed a high grade acetowhite lesion confined to the ectocervix. There were abnormal vessels present inferior to the external os. Which option or options would you recommend for further management?

Answer: __________

How confident do you feel about your answer?

I---I

not at all confident very confident

Case 9. Patient has referral cytology of HSIL, her colposcopy was satisfactory and revealed a high grade acetowhite lesion confined to the ectocervix. There were abnormal vessels present inferior to the external os. The patient is 15 weeks pregnant. Which option or options would you recommend for further management?

Answer: __________

How confident do you feel about your answer?

I---I

not at all confident very confident

Case 10. Patient has referral cytology of HSIL, her colposcopy was satisfactory and revealed a high grade acetowhite lesion confined to the ectocervix. The patient is 15 weeks pregnant. Which option or options would you recommend for further management?

Answer: __________

How confident do you feel about your answer?

I---I

not at all confident very confident

Thank you for your participation!
